# TEPA impregnation of electrospun carbon nanofibers for enhanced low-level CO_2_ adsorption

**DOI:** 10.1186/s40580-020-0217-y

**Published:** 2020-02-17

**Authors:** Jie Wang, Adedeji Adebukola Adelodun, Jong Min Oh, Young Min Jo

**Affiliations:** 1 Department of Applied Environmental Science, Kyung Hee University, 1732, Deogyeong-daero, Giheung-gu, Yogin-si, Gyeonggi-do 17103 Republic of Korea; 2 Centre for Renewable Energy Technology (CRET), The Federal University of Technology, P.M.B. 704, Akure, Nigeria

**Keywords:** Low CO_2_ capture, Physical activation, Tetraethylenepentamine (TEPA), HNO_3_ oxidation, Surface chemistry

## Abstract

The CO_2_ adsorption selectivity of plain activated carbon nanofibers (ANF) is generally low. For enhancement, nitrogen functionalities favorable for CO_2_ adsorption are usually tethered to the ANF. In the current study, we adopted chemical impregnation using 0.5 wt% tetraethylenepentamine (TEPA) solution as an impregnant. To enhance the impregnation of TEPA further, preliminary oxidation of the nanofibers with 70% HNO_3_ was conducted. The effects of HNO_3_ and TEPA treatments on the modified ANFs were investigated for physical (using N_2_ monosorb, thermogravimetric analyzer, scanning electron microscopy) and chemical (X-ray photoelectron spectrometer) changes. From the results, we found that although TEPA impregnation reduced the specific surface area and pore volume of the ANFs (from 673.7 and 15.61 to 278.8 m^2^/g and 0.284 cm^3^/g, respectively), whereas the HNO_3_ pre-oxidation increased the number of carboxylic groups on the ANF. Upon TEPA loading, pyridinic nitrogen was tethered and further enhanced by pre-oxidation. The surface treatment cumulatively increased the amine content from 5.81% to 13.31%. Consequently, the final adsorption capacity for low (0.3%) and pure CO_2_ levels were enhanced from 0.20 and 1.89 to 0.33 and 2.96 mmol/g, respectively. Hence, the two-step pre-oxidation and TEPA treatments were efficient for improved CO_2_ affinity.

## Introduction

Besides the highest contributor to anthropogenic global warming, CO_2_ could also be harmful at relatively low concentrations, especially in confined indoor spaces, depending on whether they are, stationary (such as offices, homes, and subway stations) or mobile environments (such as cars, airplane, and submarines) [1, 2]. Therefore, the control of indoor CO_2_ at 1,000 ppm or higher concentrations [the limit set by the Environmental Protection Agency (EPA)] require efficient technologies for safe indoor activities with a long duration [3].

In the last two decades, indoor air quality (IAQ) researchers have intensified efforts to improve the adsorption of elevated levels of indoor CO_2_. The use of amine-based or amine-functionalized adsorbents is popular among such research approaches [4]. Usually, these adsorbents are fabricated using grafting or the impregnation method [5]. Some researchers have confirmed that both impregnation and grafting are effective for increasing the CO_2_ adsorption capacity of carbon-based sorbents [6–8]. Furthermore, Na Rao et al. [9] compared the two methods and their results showed that impregnation is superior to grafting with respect to amine-loading efficiency and eventual CO_2_ adsorption capacity. Impregnation of amines is usually a wet process during which amines are physically adhered onto a support via non-covalent attachment to improve the affinity of the support for a target adsorptive [10]. For example, the effects of three types of organic amines (i.e., diethylenetriamine, triethylenetetramine, and tetraethylenepentamine (TEPA)) on CO_2_ adsorption capacity has been reported [11]. The results showed that if TEPA was used as a chemical impregnant for a solid support, it could improve CO_2_ adsorption of the support.

TEPA is a linear molecule bearing five amine groups per molecule: two primary amines (RNH_2_) and three secondary amine (R_2_NH) groups. The mechanism by which primary amine and secondary amine react with CO_2_ are expressed in Eqs. (1) and (2), respectively [5]:

1 $${\text{C}}{{\text{O}}_{\text{2}}} + {\text{ 2RN}}{{\text{H}}_{\text{2}}} \leftrightarrow {\text{ RN}}{{\text{H}}_{\text{3}}}^ + + {\text{ RNHCO}}{{\text{O}}^ - }$$

2 $${\text{C}}{{\text{O}}_{\text{2}}} + {\text{ 2}}{{\text{R}}_{\text{2}}}{\text{NH }} \leftrightarrow {\text{ }}{{\text{R}}_{\text{2}}}{\text{N}}{{\text{H}}_{\text{2}}}^ + + {\text{ }}{{\text{R}}_{\text{2}}}{\text{NCO}}{{\text{O}}^ - }$$

However, to achieve optimum wet impregnation, adequate knowledge of certain properties of the support such as the specific surface area, pore size, pore structure, and surface pH is essential. In general, a porous support with a relatively high specific surface area and large pore volume has a good potential for organic amine immobilization [11].

In this study, various activated nanofibers (ANFs) were prepared by either physical or chemical activation. Further, all the ANFs were impregnated with TEPA solution. To improve the TEPA loading, some ANFs were pre-oxidized with nitric acid solution prior to impregnation.

## Experimentals

### Consumable materials

Polyacrylonitrile (PAN, M.W. = 150,000 mol/g), 70% nitric acid, and tetraethylene pentamine (TEPA, Reagent grade) were purchased from SIGMA-ALDRICH, Co. in Korea.* N*,*N*-dimethylformamide (DMF), potassium hydroxide, and ethanol solution were procured from DAEJUNG CHEMICALS & METALS CO., Seoul, Korea. All of the reagents were used as-received.

### Preparation of PAN-based carbon nanofibers

First, two types of PAN solutions were prepared in weight ratios: (i) PAN/DMF solution (PAN:DMF = 1:9) for physical activation and (ii) KOH/PAN/DMF solution (PAN:DMF:KOH = 1:9:0.01–0.05) for chemical activation. Each polymer solution was electrospun at an inject velocity of 1.5 mL/h under 18 kV. The electrospun nanofiber was stabilized in an oven at 200 °C for 4 h before cooling to room temperature. Then, cooling it to room temperature, it was transferred into a tubular quartz reactor and heated at a ramping rate of 5 °C/min to 800 °C under N_2_ flow that was fed at 200 cm. Samples prepared with weight ratios (i) were activated for 15, 30, 60 and 90 min, and denoted as 15-ANF, 30-ANF, 60-ANF, and 90-ANF, respectively, whereas those prepared with weight ratios (ii) were activated for 15 min, and similarly denoted as 0.01-ANF, 0.03-ANF, and 0.05-ANF. Furthermore, 0.5 wt% TEPA solution in absolute ethanol was prepared as an impregnating solution. Then, 100 mg of each sample was added to the solution, and stirred for 6 h at room temperature. Afterwards, the samples were evaporated to dryness in an oven. However, some of the ANFs were pre-oxidized with 70% HNO_3_ solution at 80 °C for 1 h before TEPA impregnation. The HNO_3_ pre-treatment was preferably carried out below 100 °C for no more than 2 h [12]. Aside from ensuring a complete surface treatment of the fibers, the depth of the ravines increased and the fiber matrix remained uncorroded under those conditions.

### Characterization of nanofiber adsorbent

The specific surface area (S_BET_) and porosity of all the ANFs were measured by N_2_ adsorption at 77 K using a surface analyzer (Belsorp mini II, BEL, Tokyo, Japan) at relative pressures (p/p_0_) of 0–1. To pre-clean the samples prior to measurement, outgassing under N_2_ flow for 3 h at 100 °C was carried out. The equation based on BET theory (Brunauer–Emmett–Teller) was used to calculate the S_BET_, whereas the mesopore volume (V_meso_) and micropore volume (V_micro_) were obtained using Barrett–Joyner–Halenda (BJH) and MP-plot [5]. The total pore volume (V_total_) was analyzed at a relative pressure of 0.99.

The thermal stability of the adsorbents was investigated with the aid of a thermogravimetric analyzer (TGA N-1500, Scinco, USA). Each sample was heated from room temperature to 400 °C at 10 °C/min heating rate under N_2_ atmosphere. The surface morphology and feature of the samples were observed by field emission scanning electron microscopy (FE-SEM, LEO SUPRA 55, Germany) with the magnification of 50,000×. It was coupled with an energy-dispersive X-ray spectroscopy (EDS) for simultaneous elemental distribution analysis. In details, the changes in the surface chemical speciation, especially of the nitrogen functional groups, were investigated using X-ray photoelectron spectroscopy (XPS, K-Alpha, Thermo Scientific, USA).

### Measurement of CO_2_ adsorption capacity

**Fig. 1:**
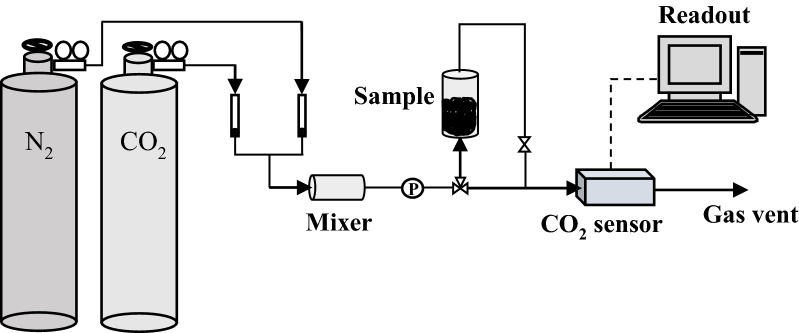
A schematic diagram of the low-level CO_2_ adsorption test set-up

The adsorption capacity of the adsorbents for pure CO_2_ was obtained using a monosorb instrument. The measurement was carried out under pressures ranging from vacuum to ambient (i.e., p/p_0_ = 0–1) at 273 K. Furthermore, the selective adsorption capacity for 0.3% CO_2_ (99.999%, in binary mixture with 99.99% N_2_) was examined using a lab scale set-up as shown in Fig. 1. The 0.3% (3000 ppm) CO_2_ represents the mean concentration of the gas commonly found in indoor spaces [13]. A CO_2_ detector (SenseAir, Sweden), equipped with a non-dispersive infrared (NDIR) sensor was used to monitor the CO_2_ concentration in the exiting gas stream. The same set-up was used for adsorption regeneration exercise carried out at 100 °C.

## Results and discussion

### Physical properties of the ANFs

Table 1 shows the textural characteristics of all of the ANF samples. We observed that the S_BET_ and V_total_ of ANFs were enhanced by either activation time or the quantity of the activation reagent. However, after impregnation with TEPA, the S_BET_ and V_total_ were reduced proportionately, an observation that is consistent with those of other studies [11, 14]. In particular, micropores of the chemically activated ANFs undetectable because the percolation of the TEPA solution blocked the tiny pores. On the contrary, the microporosity of the ANFs derived by physical activation increased after impregnation, probably due to the thermal shrinking of boundary mesopores. Since the kinetic diameter of CO_2_ is 0.33 nm, a factor that is significant to its physisorption, the proportion of microporosity is essential to the adsorption of the gas. Therefore, we inferred that ANFs prepared by physical activation are more suitable for surface modification by alkaline solution impregnation, which would improve the chemisorption of the CO_2_ molecules.

**Table 1:** Surface textural and porous properties of all of the ANF samples

Sample	S_BET_ (m^2^/g)	V_total_ (cm^3^/g)	V_meso_ (cm^3^/g)	V_micro_ (cm^3^/g)	V_micro_/V_total_ (%)
Physically activated ANF					
15-ANF	212.2	7.201	7.113	0.083	1.15
15-ANF-TEPA	60.98	0.490	0.487	0.010	2.04
30-ANF	308.4	7.882	7.802	0.137	1.74
30-ANF-TEPA	100.7	0.466	0.364	0.010	2.15
60-ANF	673.7	15.61	15.50	0.254	1.63
60-ANF-TEPA	249.2	0.207	0.190	0.047	22.5
60-ANF-HNO_3_	583.7	0.463	0.295	0.229	49.4
60-ANF-HNO_3_-TEPA	278.8	0.284	0.264	0.052	18.3
90-ANF	839.4	27.50	27.53	0.301	1.09
90-ANF-TEPA	113.0	0.246	0.170	0.015	6.01
Chemically activated ANF					
0.01-ANF	84.14	0.055	0.048	0.009	16.36
0.01-ANF-TEPA	26.64	0.040	0.046	ND	ND
0.03-ANF	184.3	0.112	0.108	0.025	22.51
0.03-ANF-TEPA	65.60	0.720	0.724	ND	ND
0.05-ANF	469.1	0.347	0.191	0.188	54.30
0.05-ANF-TEPA	50.17	1.445	1.393	ND	ND

*ND* not-detected

**Fig. 2:**
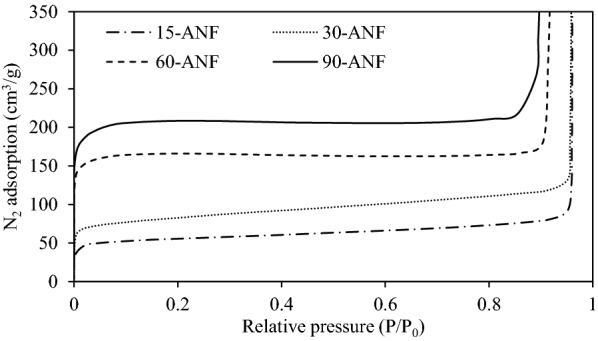
N_2_/77 K isotherm of physically activated ANFs

The N_2_ adsorption isotherms of a part of physically activated ANFs are compared in Fig. 2. All the isotherms followed a typical behaviour of Type I between 0 and 0.8 of the relative pressure, indicating single-layer reversible adsorption. Type I adsorption frequently occurs on microporous solids. The initial adsorption capacity is relatively large with the change of relative pressure, and then the adsorption capacity will not change greatly with the increase of relative pressure [15]. However, as P/Po approached 1, the adsorption capacity increased infinitely. This was caused by multiple molecular layers (physisorption) of the sorptive formed at various thicknesses on the solid surface, leading to the formation of Type II adsorption isotherm. Therefore, it can be seen that the ANFs prepared has both inner micropores and outer mesopores well developed.

To further enhance TEPA loading on the ANFs at the same impregnation concentration, the ANFs were oxidized with 70% HNO_3_ solution for 1 h prior to TEPA impregnation. The HNO_3_ oxidation formed crevasses and pits by eroding the graphene layer primarily from the basal planes of carbonaceous materials [16]. After HNO_3_ oxidation, the surface became irregular and there were more oxygen-based active groups such as carboxyl, carbonyl and ether groups tethered onto the ANFs [17–19]. The presence of these active groups were advantageous for the attachment of TEPA onto the adsorbent surface via a displacement reaction [20, 21].

The optimization of HNO_3_ pre-oxidation was conducted with 60-ANF because the textural quality (S_BET_ and V_micro_/V_total_) of the 60-ANF-TEPA sample was found best in the TEPA-impregnation step. After pre-oxidation, the V_total_ of 60-ANF decreased significantly from 15.61 to 0.463 cm^3^/g. This was a result of mesopore shrinkage, resulting in a large increase in the proportion of micropore volume (V_micro_/V_total_) from 1.63 to 49.4%. The V_micro_/V_total_ has been reported to be the driving force for selective adsorption of CO_2_ [22]. Despite the significant improvement in this property, a slight decrease in the S_BET_ of the 60-ANF-HNO_3_ sample was observed, which was attributed to the collapse of some mesopores. From the TEPA impregnation results of the adsorbent (before and after oxidation), the pre-treated 60-ANF-HNO_3_-TEPA sample exhibited superior specific surface area and pore volume. However, the impregnation of TEPA depreciated the microporosity of the adsorbent (from 60-ANF-HNO_3_ to 60-ANF-HNO_3_-TEPA), i.e., a much superior microporosity was developed compared to that of 60-ANF. Finally, the pre-oxidized TEPA-doped ANFs showed more superior properties than those that were chemically activated.

**Fig. 3:**
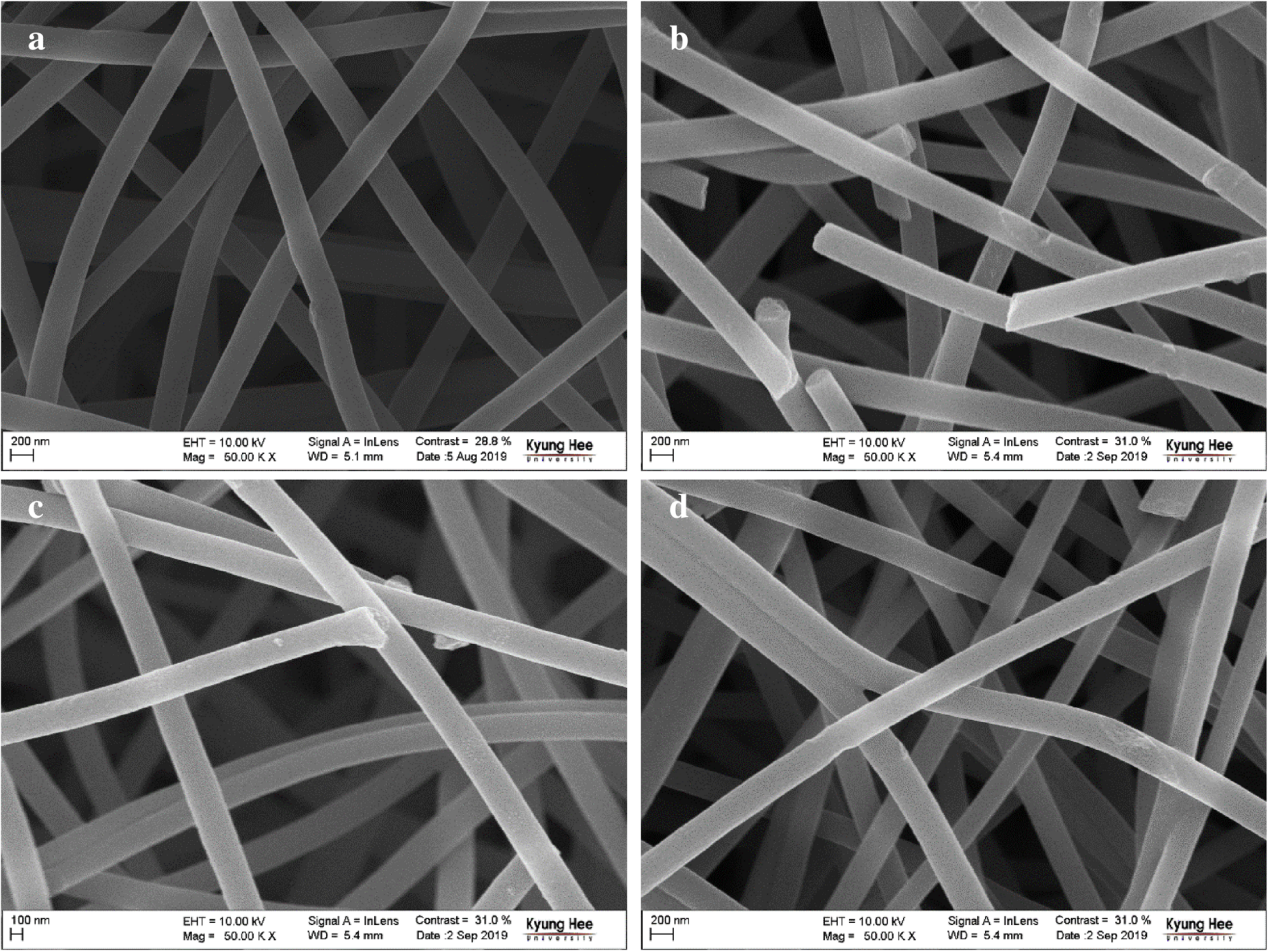
FE-SEM images of **a** 60-ANF; **b** 60-ANF-TEPA; **c** 60-ANF-HNO_3_; **d** 60-ANF-HNO_3_-TEPA

The surface morphology of fibers is shown in the FE-SEM micrographs of ANFs (Fig. 3), produced at 50,000× magnification with 10 kV accelerating voltage. It can be seen that various surface modified ANFs exhibited uniform size distribution of fiber thickness. However, there were obvious etching on the surface of the TEPA-modified ANFs (Fig. 3b, d), while those of 60-ANF-TEPA sample (Fig. 3b) was deformed. Generally, the structural effects of HNO_3_ pre-oxidation on the ANFs correspond with the differences in the S_BET_ and V_total_ of 60-ANF-HNO_3_, which were higher than those of 60-ANF-TEPA and 60-ANF-HNO_3_-TEPA (Table 1).

**Fig. 4:**
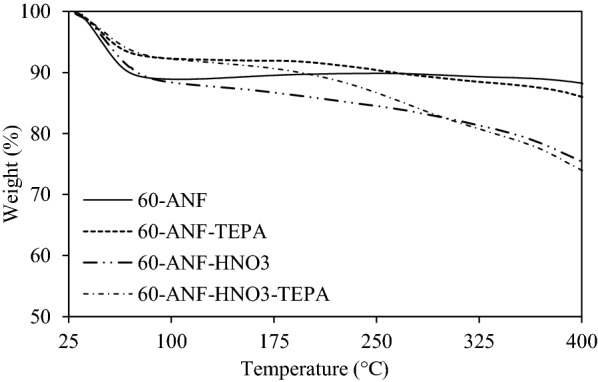
Thermograms showing slopes that are indicative of the thermal stability of ANFs

To understand the thermal stability of modified ANFs, we performed a thermogravimetric analysis on the samples. The thermograms obtained are shown in Fig. 4. All of the samples showed different rates of weight loss as the volatile organics and moisture content were eluted below 100 °C. TEPA-treated samples evinced a less steep slope than those that were untreated, indicating more refractiveness of the contents introduced by TEPA treatment. Between 100 and 400 °C, the 60-ANF sample showed the most impressive thermal stability, as a steady plateau was observed. Such steadiness could be attributed to the homogeneity of the fibers, devoid of foreign dopants in the matrix as can be seen in Fig. 3a. However, the thermogram of pre-oxidized fibers exhibited steeper depreciation because the oxidized fiber surfaces were more easily decomposed under an increasing temperature [20]. The strong acidic functionalities such as carboxylic, anhydrides and lactones tend to be decomposed at low temperatures, while the weak acidic functionalities such as carbonyl, phenol and quinone are dissipated at high temperature [23, 24]. The steady weight loss of TEPA impregnated samples (60-ANF-TEPA and 60-ANF-HNO_3_-TEPA) began at about 180 °C. From the difference in their thermograms, we inferred that chemical modification was most pronounced on the surface of the pre-oxidized 60-ANF-HNO_3_-TEPA sample.

### Chemical properties of the ANFs

Through high resolution XPS analysis of the samples, the major peaks of the scan spectra determined to be due to the C_1s_, O_1s_, and N_1s_ photoelectrons. Table 2 lists the surface elemental compositions and atomic ratios of optimized 60-ANF samples. As expected, the HNO_3_ treated sample (60-ANF-HNO_3_) showed higher O_1s_/C_1s_ ratios (14.75%) and N_1s_/C_1s_ ratios (7.95%) than the unoxidized precursor (60-ANF). With HNO_3_ treatment, the amount of O_1s_ and N_1s_ increased significantly, while the proportion of C_1s_ decreased due to surface eroding of the graphene layers, attributed to wet oxidation [25]. With the same impregnant concentration, the oxidized 60-ANF-HNO_3_-TEPA sample showed a higher N_1s_/C_1s_ ratios (17.19%) than that of the 60-ANF-TEPA (12.81%) sample, indicating that the HNO_3_ oxidation treatment improved the loading of TEPA.

**Table 2:** The XPS-derived elemental compositions and atomic ratios of 60-ANF-based samples

Element (%)	Elementary composition (%)			Atomic ratio (%)	
	C_1s_	O_1s_	N_1s_	O_1s_/C_1s_	N_1s_/C_1s_
60-ANF	89.02	5.16	5.82	5.80	6.54
60-ANF-TEPA	84.20	5.01	10.79	5.95	12.81
60-ANF-HNO_3_	81.50	12.02	6.48	14.75	7.95
60-ANF-HNO_3_-TEPA	77.41	9.28	13.31	11.99	17.19

**Fig. 5:**
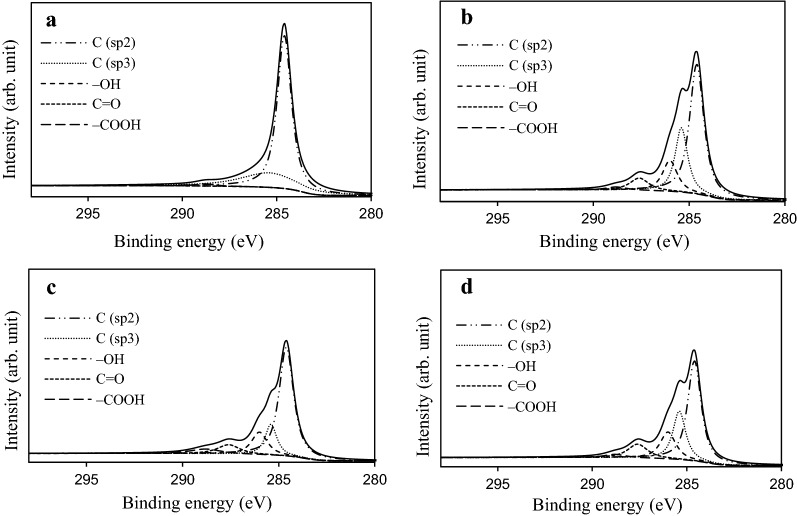
The high resolution of the deconvoluted XPS C_1s_ spectra of the ANF samples: **a** 60-ANF; **b** 60-ANF-TEPA; **c** 60-ANF-HNO_3_; and **d** 60-ANF-HNO_3_-TEPA

To better understand the chemical speciation of C and N on the selected samples, deconvolution of the XPS spectra was performed in the C_1s_ and N_1s_ regions. Figure 5 shows the best curve fit for the high resolution XPS C_1s_ spectra of all of the samples. Table 3 provides the percentage composition of the functional groups in the C_1s_ regime as (i.e. C in polyaromatic structures (C (sp^2^), BE = 284.6 eV) or aliphatic structures (C (sp^3^), BE = 285.4 eV): in phenolic, alcohol, ether or C=N groups (BE = 286.0 eV): in carbonyl or quinone groups (BE = 287.6 eV): and in carboxyl, lactone, or ester groups (BE = 288.8 eV) [26]. With either HNO_3_ or TEPA treatment, the appearance of –OH and C=O groups on the fiber surface resulted in a decrease in the relative proportion of C (sp^2^) and C (sp^3^). Wet oxidation often incorporates high amount of oxygen, as carboxylic and phenolic functionalities on the carbon surface [20]. As expected, upon HNO_3_ oxidation (60-ANF-HNO_3_), the amount of –COOH groups increased significantly. However, the content of –COOH groups in the TEPA impregnated 60-ANF-TEPA and 60-ANF-HNO_3_-TEPA samples decreased by 0.4% and 2.87% respectively, compared to that of 60-ANF and 60-ANF-HNO_3_. Of the two possible types of acidic reactive groups present on the surface (phenolic –OH and carboxylic –COOH), only the carboxyl groups could undergo a reaction with TEPA because both phenolic and aliphatic hydroxyl groups are essentially inert toward primary and secondary amino groups [27]. This result indicated the successful substitution reaction of the acidic oxygen functionalities by the N-bearing groups from TEPA.

**Table 3:** Quantitative results of the fits of the XPS C_1s_ region, given in % of total intensity

Sample	286.4 C (sp^2^)	285.4 C (sp^3^)	286.0 –OH	287.6 C=O	288.8 –COOH
60-ANF	71.64	26.56	–	–	1.80
60-ANF-TEPA	58.31	24.03	10.37	5.89	1.40
60-ANF-HNO_3_	59.74	13.52	13.54	7.90	5.30
60-ANF-HNO_3_-TEPA	49.61	22.58	15.43	9.94	2.43

**Fig. 6:**
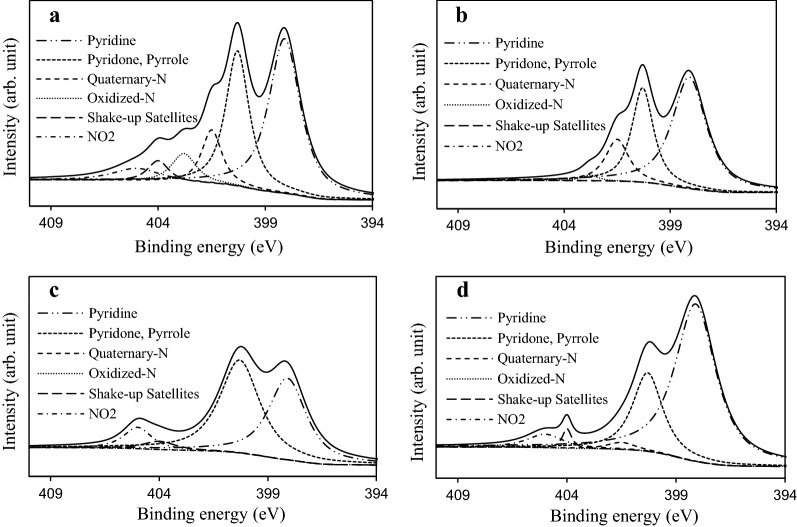
The high resolution of deconvoluted XPS N_1s_ spectra of the ANF samples: **a** 60-ANF; **b** 60-ANF-TEPA; **c** 60-ANF-HNO_3_; and **d** 60-ANF-HNO_3_-TEPA

The nature and amount of the N species tethered by the amination were investigated. Figure 6 shows the best curve fit for the high resolution XPS N_1s_ spectra of all samples, while Table 4 lists the percentage of nitrogen-containing functional groups. The primary surface nitrogen groups found on the ANFs were pyridine-type (BE = 398.1 eV), pyridone and pyrrole (BE = 400.3 eV), quaternary-N (BE = 401.5 eV), oxidized-N (BE = 402.8 eV) [28], and chemisorption of NO_2_ at 405 eV [14]. Pyridinic and pyridine/pyrrole N were the two dominant N-groups found on the 60-ANF. Both accounted for about 74% of the total N, while the other groups were present in minor to trace quantities. However, upon the impregnation of TEPA (60-ANF-TEPA), there was a significant increase in the pyridinic and quaternary N-groups, possibly derived from the total conversion of shake-up satellites and adsorbed NO_2_. Such observation which implied that the linear TEPA resulted in the increase of pyridine-like structures or six-member or five-member rings [14]. As expected, HNO_3_ oxidation evinced lowering of basic N-groups (pyridines), while the least basic groups (shake-up satellites and adsorbed NO_2_) were re-introduced to the ANFs. Furthermore, final TEPA amination of the pre-oxidized samples (60-ANF-HNO_3_-TEPA) ensured that the quantity of the desired pyridinic N was optimized, while the unwanted shake-up satellites and adsorbed NO_2_ groups were significantly lowered. We concluded that although TEPA is a viable amination agent, it also incorporates refractory quaternary-N while expunging unwanted N groups. In addition, HNO_3_ was confirmed as an excellent pre-oxidant for TEPA amination of ANFs, as it improved the tethering of preferred pyridine groups which are favorable for selective adsorption of CO_2_ over N_2_ [29].

**Table 4:** Quantitative results of the deconvoluted XPS N_1s_ spectra values given in % of total intensity

Sample	398.1 eV Pyridine	400.3 eV Pyridone, Pyrrole	401.5 eV Quaternary-N	402.8 eV Oxidized-N	404 eV Shake-up satellites	405 eV NO_2_
60-ANF	44.98	29.07	9.84	6.54	4.03	5.54
60-ANF-TEPA	55.48	29.30	13.92	1.30	–	–
60-ANF-HNO_3_	40.38	48.34	-	–	3.46	7.82
60-ANF-HNO_3_-TEPA	66.88	24.25	2.02	–	1.86	4.99

**Fig. 7:**
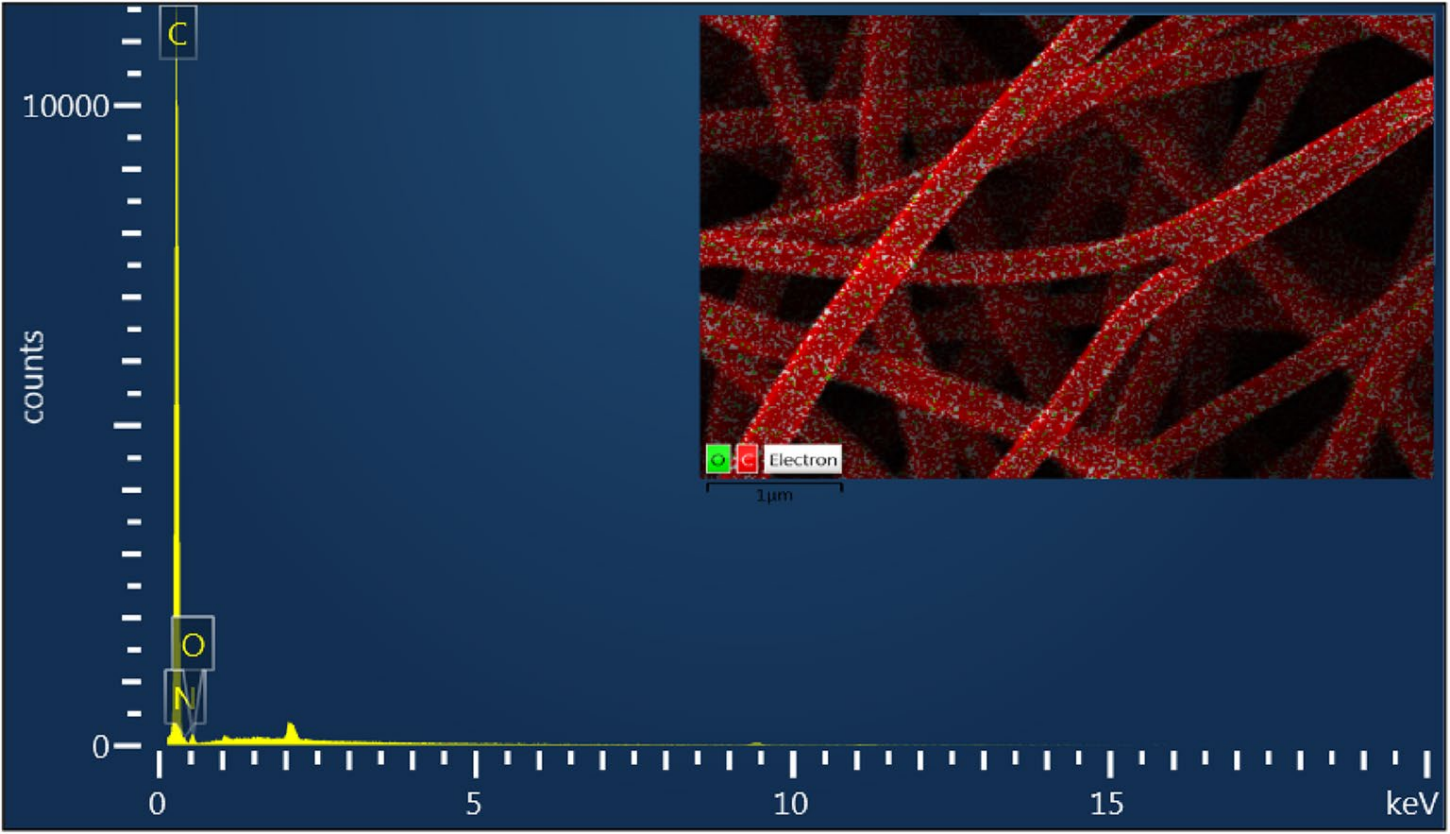
The EDS spectrum of the sample 60-ANF-HNO3-TEPA (inset: EDS layered image)

To complement the elemental distribution analysis carried out with the XPS, an EDS analysis was performed on the optimized sample (60-ANF-HNO_3_-TEPA). As shown in the Fig. 7, the EDS spectrum identified only a trace amount of N on the sample surface. Also, finite but uniform distribution of carbon and oxygen was observed. This was because XPS analysis is more sensitive than EDS, with a minimum detection concentration of 0.1%, although at the analysis depth of 10 –20 nm only, while EDS analysis occurs bulk measurement that reaches micron level [30, 31]. Since the impregnated N-groups were populated on the surface of the adsorbent, the EDS analysis could only detect a lesser percentage compared to those of the XPS. Still, both measurements give comparatively similar results when compared in proportion.

## Adsorption and regeneration of nanofiber adsorbents

### CO_2_ adsorption capacities of the ANFs

Table 5 shows the adsorption capacities for 0.3% and 100% CO_2_ of all the test samples. We found that with either an increase in the activation time or the quantity of the activation reagent, the CO_2_ adsorption capacity increased for both levels. This result is fundamentally true because gas adsorption is positively correlated with both the specific surface area and microporosity [32]. Although the S_BET_ of chemically activated ANFs was smaller than that of physically activated ANFs, the adsorption capacities of the former for pure CO_2_ were still significantly improved. In regards to the 0.3% CO_2_ test feed (Table 5), physically activated ANFs exhibited significantly higher adsorption capacities than their counterparts. We ascribed this result to the larger specific surface area induced by physical activation, which, in turn, favored TEPA impregnation and eventually CO_2_ capture. In addition, the pre-oxidized 60-ANF-HNO_3_-TEPA sample had higher amino loading (Table 2 and Fig. 4), resulting in the adsorption of 0.3% CO_2_, which was about quadruple that of not pre-oxidized 60-ANF-TEPA.

**Table 5:** CO_2_ adsorption capacities (q) of all ANF samples prepared in this work

Sample	CO_2_ adsorption (mmol/g)	
	0.3%	100%
Physically activated ANF		
15-ANF	0.107	1.89
15-ANF-TEPA	0.034	0.65
30-ANF	0.151	2.10
30-ANF-TEPA	0.119	0.97
60-ANF	0.203	2.84
60-ANF-TEPA	0.087	1.48
60-ANF-HNO_3_	0.121	1.98
60-ANF-HNO_3_-TEPA	0.334	2.96
90-ANF	0.238	2.80
90-ANF-TEPA	0.117	1.00
Chemically activated ANF		
0.01-ANF	0.021	1.96
0.01-ANF-TEPA	0.015	0.33
0.03-ANF	0.028	2.67
0.03-ANF-TEPA	0.013	0.64
0.05-ANF	0.096	2.68
0.05-ANF-TEPA	0.020	0.41

Table 6 shows a comparison list of the CO_2_ adsorption capacities of various adsorbents from open literatures. For all the sorbents, the higher S_BET_ and V_micro_/V_total_ were, the higher the adsorption capacity of pure CO_2_ was. In our study, an increase in amino group content on the surface of the modified samples also led to a relatively high CO_2_ adsorption capacity. However, as most published works have focused on pure and flue gas CO_2_ levels, our work is unique in that we attempted to improve the CO_2_ adsorption selectivity from indoor low levels.

**Table 6:** Comparison of the CO_2_ adsorption capacities (q) of the current work with those from literature

Support	Modification chemicals	S_BET_ (m^2^/g)	V_micro_/V_total_ (%)	CO_2_ feed level	CO_2_ adsorption (mmol/g)	References
ANF	–	300	48	100%	2.74	[33]
	Urea 1:4	542	92	100%	2.98	[34]
Commercial ACF	10 wt% TEPA	1051	73	15%	0.50	[14]
CNTs	APTS	15.87	53	15%	0.98	[35]
ACF	HN_3_ gas	1293	85	0.3%	0.40	[36]
AC	HN_3_ gas	1251	–	10%	0.63	[37]
				0.3%	0.15	
ANF	0.5 wt% TEPA	278.8	18.3	0.3%	0.33	This work
				100%	2.96	

### Regeneration capacity of the ANFs

**Fig. 8:**
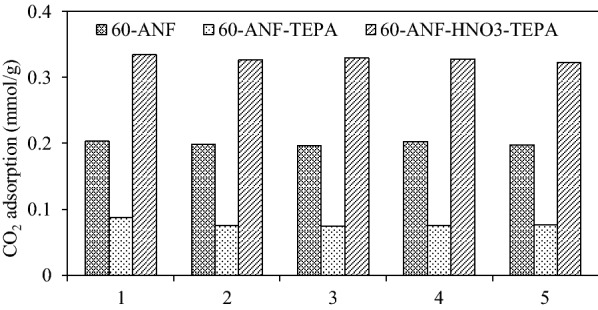
Adsorption capacity of carbon dioxide in five cycles

The regeneration of our representative samples for each treatment were examined by a simple temperature programmed desorption (TPD) using the TGA. The desorption temperature was set at 100 °C under N_2_ atmosphere. It has been confirmed that the desorption of CO_2_ form CO_2_–TEPA interaction is likely occurred in two stages: (i) slow transport via the decomposition of primary ammonium–carbamate species, followed by (ii) rapid desorption of primary and secondary ammonium–carbamate species at 100 °C. At 100 °C, the ammonium–carbamate species was eliminated, which liberated most of the adsorbed CO_2_ [38]. Figure 8 shows the 5-cycle regeneration adsorptions of CO_2_ (3000 ppm). Quantitatively, the adsorption capacity of sample 60-ANF, 60-ANF-TEPA and 60-ANF-HNO_3_-TEPA decreased mildly by 2.62%, 11.49% and 3.58% respectively, after 5 cycles. The regeneration performance of the TEPA impregnated samples was relatively weak, which may be caused by the thermal-induced elution of some TEPA molecules from the surface.

## Conclusions

Nanofibers were prepared by electrospinning before physical or chemical activation to obtain activated nanofibers (ANFs) with varying structural and chemical characteristics. All of the ANFs were impregnated with a TEPA solution of varying amounts. By examination, we found that the ANFs that were physically activated were more suitable for TEPA impregnation because of the enhanced specific surface area and porosity. After TEPA impregnation, the specific surface area and total pore volume of the adsorbents decreased significantly. After the HNO_3_ pre-oxidation treatment, the amine loading improved the incorporation of useful, basic pyridinic nitrogen, whereas the least basic N functionalities were effectively removed or converted. Therefore, the adsorption capacity of the optimized 60-ANF-HNO_3_-TEPA for 0.3% and 100% CO_2_ increased from 0.12 and 1.89 mmol/g (by 60-ANF-TEPA) to 0.33 and 2.96 mmol/g, respectively. A 5-cycle regeneration test showed a relatively stable adsorption of thermally refreshed adsorbents for proficient reusability. Consequently, HNO_3_ pre-oxidation of TEPA-doped ANF is efficient for enhancing low-level CO_2_ adsorption from indoor spaces.

## Data Availability

Not applicable.

## Abbreviations

ANF: activated carbon nanofiber
TEPA: tetraethylenepentamine
EPA: Environmental Protection Agency
IAQ: indoor air quality
PAN: polyacrylonitrile
DMF: *N*,*N*-dimethylformamide
FE-SEM: field emission scanning electron microscopy
XPS: X-ray photoelectron spectroscopy
EDS: energy-dispersive X-ray spectroscopy
NDIR: non-dispersive infrared
ND: not-detected
IUPAC: International Union of Pure and Applied Chemistry
BE: binding energy
APTS: 3-aminopropyl-triethoxysilane
TPD: simple temperature programmed desorption
